# Basketball self-evaluation matrix: discrepancy between self-confidence and decision-making performance on psychological profiling of players

**DOI:** 10.3389/fspor.2024.1404701

**Published:** 2024-10-09

**Authors:** Mengru Liu, Anthony Kong, Newman Lau, Zeping Feng, Xi Liu

**Affiliations:** ^1^ School of Design, The Hong Kong Polytechnic University, Hong Kong, Hong Kong SAR, China; ^2^ Laboratory for Artificial Intelligence in Design, Hong Kong, Hong Kong SAR, China

**Keywords:** youth basketball players, self-evaluation, decision-making, self-confidence, psychological profiling

## Abstract

**Background:**

In basketball training, self-evaluation plays a crucial role in the decision-making and execution of movements of players. The self-evaluation of players is influenced by their perception of own basketball ability and self-confidence state. This study aimed to explore potential discrepancies between self-confidence levels of players and their decision-making performance, while also characterizing different types of players.

**Method:**

Data was collected from 20 youth basketball players who participated in a decision-making video task and a self-confidence assessment. Based on data from their self-confidence and decision-making awareness assessment, the K-means cluster analysis was used to categorize the players into different groups. Then, ANOVA and *post hoc* Scheffe test were conducted to compare these clusters.

**Results:**

The cluster analysis identified four distinct profiles of players and the results of the ANOVA and *post hoc* Scheffe tests revealed significant differences between the four clusters. The “High Self-confidence & Low Decision-making Awareness” players might display an overconfident mindset, while the “High Self-confidence & High Decision-making Awareness” players potentially demonstrated the better performance and maintained a consistent and confident attitude. The “Low Self-confidence & High Decision-making Awareness” players appeared to lack confidence and needed to foster greater faith in their abilities. Finally, the “Low Self-confidence & Low Decision-making Awareness” players required a long-term and comprehensive training program to improve their skills.

**Discussion:**

These preliminary findings informed the development of a self-evaluation matrix, designed to help coaches better understand player profiles and design tailored interventions. Moreover, this study contributes on sport calibration and enhances understanding of the behavioral and psychological states of players.

## 1 Introduction

**Figure 1:**
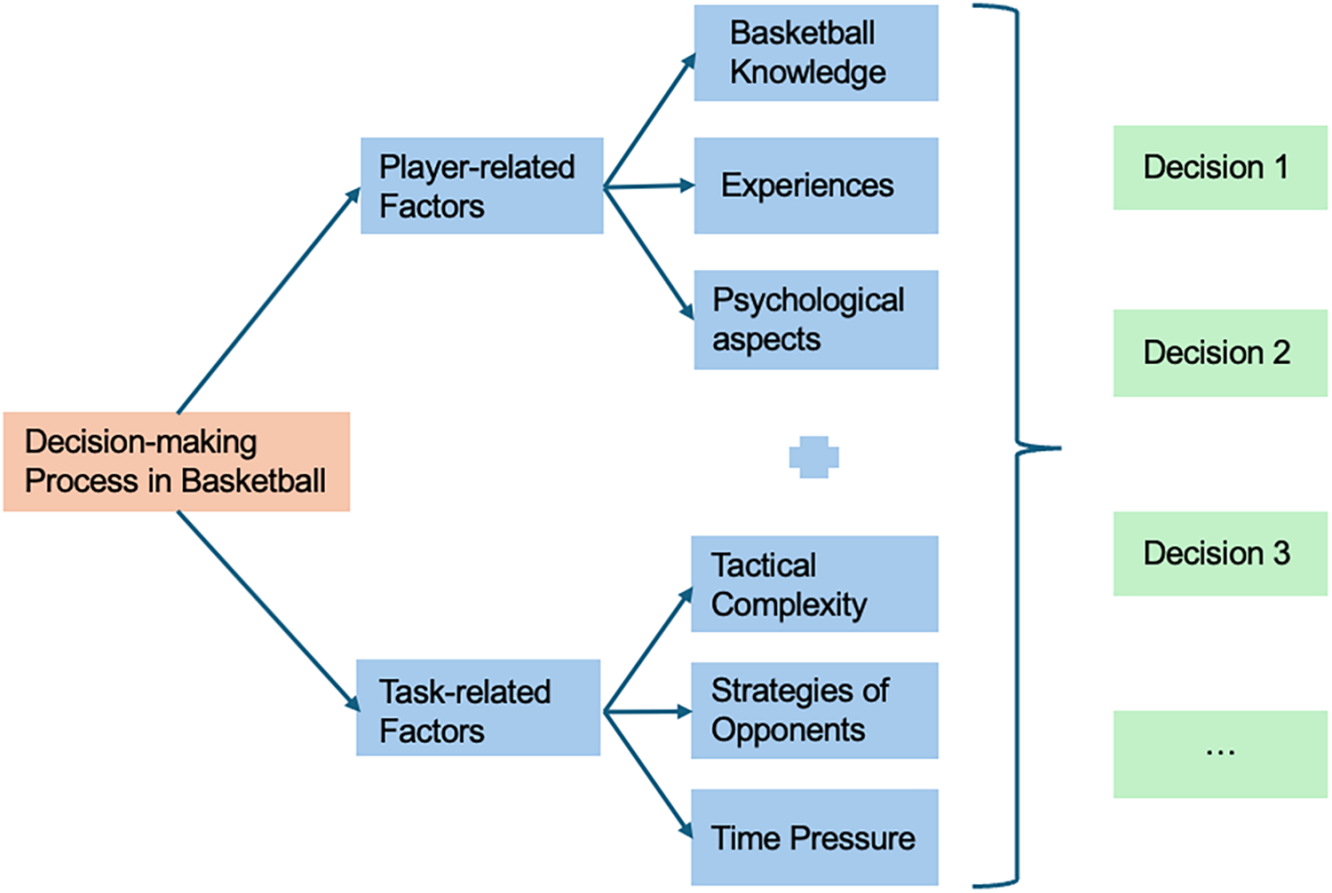
Decision-making process in basketball.

In basketball competitions, players confront challenging and constantly changing situations on the court, where decision-making emerges as a complex skill crucial for game success. This process is significantly influenced by both player-related and task-related factors (1). Player-related factors encompass basketball knowledge, experiences, and psychological aspects such as player confidence, which notably motivates quick and effective decisions during matches (1–3). The level of self-confidence in players, closely tied to their self-evaluation and self-perception, plays a pivotal role in their performance (4). Task-related factors, including tactical complexity, opponent strategies, and time pressure, also critically affect players’ ability to make decisions under stress and complexity. Therefore, the decision-making process in basketball is not determined by a single factor but rather emerges from the dynamic interplay between player-related attributes (such as psychological readiness) and the demands imposed by the game (such as tactical awareness). As illustrated in Figure 1, this interaction between the internal preparedness of players and the external challenges they face during a game forms the foundation of decision-making on the court.

Furthermore, A key element of player-related factors in decision-making process is feedback, which influences players’ self-evaluation and self-perception. When players possess the ability to accurately evaluate themselves by self-report evaluation, which comes from their own perception of performance, as well as augmented feedback provided by external sources such as coaches or teammates, they can more effectively to observe the game and adjust their mental state, leading to appropriate decision-making with confidence in various game scenarios and contributing to successful outcomes (5, 6). Conversely, when self-evaluation is not properly calibrated, either due to a lack of accurate feedback or poor self-perception, it may lead to poor decision-making and unfavorable game outcomes (7).

Various studies have indicated that a significant number of basketball players struggle with accurately assessing their abilities, resulting in decision-making rooted in exaggerated or undervalued perceptions of their skills, ultimately leading to either overconfidence or a lack of self-confidence (8, 9). Player lack of confidence causes fear, which is a known psychological predictor of inability in sports performance (10). It is worth noting that when certain team members lack self-confidence and perform inadequate basketball knowledge, the consequences extend beyond individual performance, significantly impacting team cohesion (11).

It has been observed that some players who exhibit overconfidence in basketball tend to disregard the holistic context of the game as a consequence of making impulsive decisions (12). Moreover, these players often report diminished satisfaction when fulfilling basketball-related responsibilities, as a result of inflated expectations and subpar performance outcomes (13). The exhibition of overconfidence or lack confidence by players not only yield unfavorable final game outcomes, but also exerts a substantial impact on the overall sports experience and engendered interest in basketball, thus impeding the development of potential athleticism. Researchers proposed that the inaccurate self-evaluations of players can be attributed to their insufficient understanding and mastery of current tactics and skills. As players become more familiar with the intricacies of the game, their ability to accurately assess their own capabilities improves (14). Moreover, considering the multifaceted nature of basketball and external evaluations from coaches are essential to foster more precise self-perceptions among players (15).

However, the complexity and dynamic nature of basketball make it challenging for novice players to quickly master skills and understand various tactical strategies (16). Additionally, some novice and intermediate players may struggle with inconsistent performance in games, leading to a lack of confidence and interest in basketball training (17). Experienced players who have undergone extensive training often rely on unconscious decision-making on the court, making it difficult for them to articulate their decision-making process in different tactical situations and hinder their ability to improve (18). Coaches, especially when working with large groups of players, may find it challenging to observe timely changes in their players, particularly when it comes to subtle psychological activities (19). This can result in a limited ability to provide comprehensive evaluations for players, ultimately affecting the effectiveness of training plans tailored to individual needs and talent identification (20).

Some previous research has examined the use of self-evaluation, characteristic recognition and predict tasks to assist participants in understanding their sports ability and potential better (21–23). For instance, Válková (24) analyzed the impact of self-evaluation on the acquisition of fundamental basketball skills. Cartigny and colleagues (25) identified various characteristics of dual professional athletes by assessing self-efficacy and identity. Silva and colleagues (14) investigated the relationship between self-evaluation abilities and objective performance measures. Schweitzer (26) identified the effects of unjustified confidence and overconfidence on basketball ability. These studies utilized methods such as self-evaluation questionnaire, feature recognition and observe tasks to assist participants in gaining comprehensive insights into their characteristics, including changes in psychological states. However, there is a lack of research that explores the discrepancies between decision-making awareness and self-confidence in helping players accurately identify themselves basketball abilities, as well as how these insights can assist coaches in designing training strategies and managing players effectively.

Therefore, this study examined the accuracy of self-evaluation in the context of different offensive and defensive strategies by considering the factors of decision-making awareness and self-confidence. We aimed to analyze basketball ability and explore the changes in psychological characteristics among different groups of players. The study facilitated a better understanding of the strengths and weaknesses of players through the application of self-evaluation, helping them make more informed and effective decisions on the basketball court. By classifying players into distinct characteristic groups, this study provided insights that assist coaches in better understanding and managing their players, tailoring training programs to individual needs, and potentially improving overall training efficiency. Furthermore, this study contributes to basketball research by offering a self-evaluation matrix that could guide future studies and help industry practitioners develop more targeted strategies.

## 2 Method

This study used a quantitative method that included both observational and analytical components. Specifically, the researchers collected data from 20 youth basketball players through two main assessments: a decision-making video task and a self-confidence assessment.

### 2.1 Participants

20 male basketball players were recruited from a high school basketball team in the southwest region of China. According to Creswell (27), researchers typically estimate that involving between 10 and 50 participants is an appropriate sample size for qualitative and pilot studies. The age of all players ranged from 14 to 17 years old (M age = 15.85 years, SD = 1.04). Participants were recruited through communication with the team's coach. Inclusion criteria required players to be active team members participating in regular weekly training sessions. Exclusion criteria included players with injuries or health conditions. On average, these players have been involved in basketball for approximately 2.65 years. Prior to participation, all individuals provided their informed consent after receiving detailed information about the study. Ethical approval was granted from the Institutional Review Board (IRB) of The Hong Kong Polytechnic University (Reference Number: HSEARS20230823003).

### 2.2 Instrument

#### 2.2.1 Decision-making video task and self-confidence assessment

A decision-making video task was implemented in this study to establish a tactical-oriented assessment system for basketball players. This task involved the observation of defensive and offensive situations through game videos, requiring participants to select the appropriate next movement from different options. Previous research has utilized video-feedback tasks and questionnaires to examine self-confidence levels and decision-making abilities (28, 29). For instance, Powless and colleagues (30) explored the relationship between decision-making performance and self-efficacy using decision-making task. López-Aguilar and colleagues (31) investigated the association between the decision self-efficacy and decision-making processes of players, encompassing option generation and selection, within soccer match play. Video feedback tasks have been employed to assist players in evaluating their decision-making capacities, enhancing their tactical perception, and identifying fast and intricate movement cues, as well as specific patterns within the game (32). In this study, 10 video clips showcasing offensive and defensive basketball tactics were edited and customized from a professional basketball match (33). These video simulation scenarios were designed to assess the basketball decision-making skills of the players.

To ensure the validity and reliability of the instrument, a triangulation approach was adopted, incorporating feedback from three key perspectives: experienced two basketball head coaches, five teenage players, and a researcher with basketball experience. The content validity was reviewed by head coaches, who ensured that the video scenarios and response options accurately reflected the decision-making process of players during real games. Feedback from teenage players helped confirm that the scenarios were relevant and realistic from a participant's perspective. The researcher ensured the appropriateness and practical relevance of the instrument design. Additionally, the use of standardized scoring criteria across all participants helped to enhance the reliability of the assessment, ensuring consistent evaluations and minimizing subjective scoring biases.

### 2.3 Procedure

During the experimental sessions, participants were tasked with predicting the next movement of a given basketball player and rating their confidence in their chosen answer. This task was conducted through the observation of 10 basketball tactic video clips, each with an average duration of 10.5 s. The entire experimental process was completed within a single day. Prior to the commencement of the experiment, the researchers utilized two prepared non-experimental video clips to explain the rules of the study and address any queries raised by the participants.

**Figure 2:**
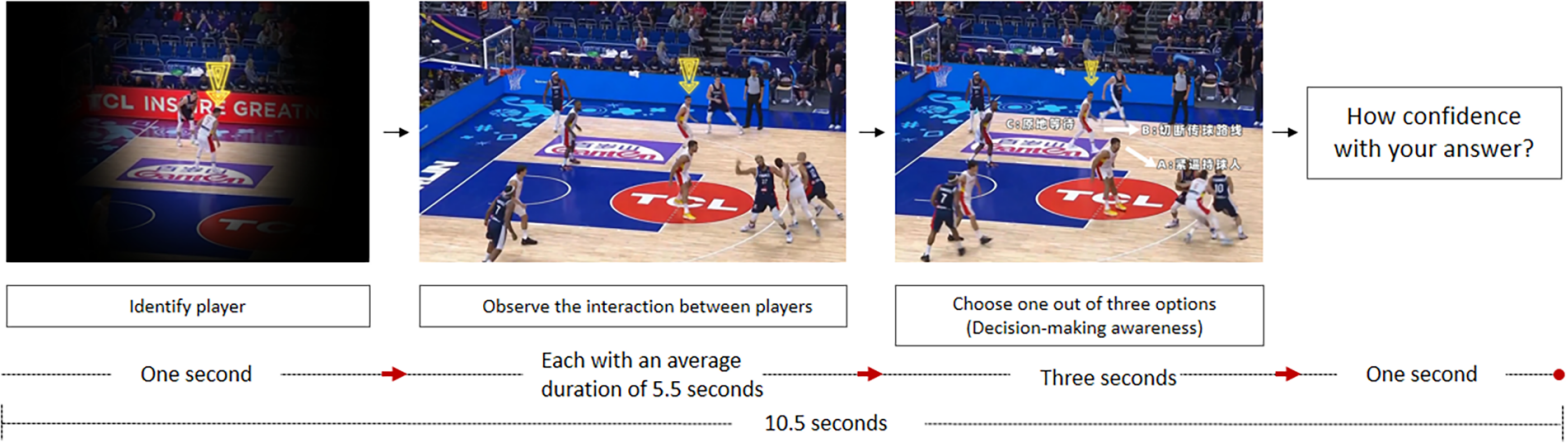
The procedure of one of the videos used during the experiment sessions.

To ensure efficient and accurate responses from the participants, as depicted in Figure 2, the video clips were meticulously designed. They included a brief masking period of one second, during which the rest of the scene was concealed, and only the basketball court position of the player to be identified was visible. Following this period, the video clip commenced, displaying the interactions between players. Subsequently, the video paused, and three possible options were presented to the participants. They were then given three seconds to select the next movement they believed was appropriate for the player. After making their decision for each video clip, participants were asked to rate their confidence level in their decision using a 4-point Likert scale, ranging from “not at all confident” to “completely confident”.

In this study, the scoring for each of the three options presented for each video clip was determined through the review process involving two head coaches. The best action was assigned a score of 3, an acceptable action received a score of 2, and a suboptimal action obtained a score of 1. Employing this approach allowed the researchers to examine the potential discrepancy between the self-confidence of players and their actual decision-making ability. This method effective in identifying various psychological characteristics of the players, such as a tendency towards overconfidence or a lack of confidence, as well as their decision-making abilities.

## 3 Data analysis

The data analysis consisted of a K-Means cluster analysis (performed in R version 4.3.1 and R studio), and an analysis of variance (ANOVA) and *post hoc* scheffe analysis (performed in IBM SPSS statistics package version 27.0). The K-means cluster analysis was utilized due to its simplicity, fast convergence, and its suitability for smaller datasets (34). Upon conducting the study, data were collected from 20 players on four variables: decision-making awareness in offense, Self-confidence in offense, decision-making awareness in defense, and Self-confidence in defense. K-means cluster analysis was used to categorize the players based on their characteristics of decision-making awareness and Self-confidence in offensive and defensive tactics. Subsequently, ANOVA and *post hoc* scheffe analysis were utilized to conduct meticulous between-group comparisons, thus providing comprehensive insights into the unique characteristics exhibited by players with each cluster.

### 3.1 Clustering players by decision-making abilities and self-confidence levels

#### 3.1.1 *K*-means cluster analysis on player attributes

The *k*-mean is an intuitive and easy to understand clustering algorithm, suitable and efficient for practical applications, and the algorithm is computationally faster when working with data, especially if the data dimensions are low. The *K*-means clustering analysis method is employed to effectively group players based on their shared attributes, thereby forming clusters characterized by high similarity. It represents an iterative algorithm employed to partition datasets into *K* non-overlapping clusters, with each cluster being effectively characterized by its centroid. The clustering procedure begins with the selection of *K* initial centroids by users that determines the desired number of clusters (34). Subsequently, each data point is assigned to the closest centroid, thus delineating individual clusters based on proximity. The centroids of these clusters are updated iteratively in response to the data points assigned to each cluster. This iterative process persists until no further changes in point-to-cluster assignments occur, signifying convergence. *K*-means can be expressed by an objective function that aims to minimize the distances between data points and their corresponding cluster centroids, as shown in the Equation:

$$\underset{\left\{m_{k}\right\},1\leq k\leq K}{\min}\;\overset{K}{\underset{k=1}{\sum }}\;\underset{xC_{k}}{\sum }\;\pi_{x}\mathrm{dist}\left(x,m_{k}\right)$$

The function involves a double summation, with the outer sum iterating over *K* clusters (k=1toK) and the inner sum over data points within each cluster ($C_{k}$).The membership weight ($\pi_{x}$) of a data point *x* influences the position of centroid. By minimizing distances, the algorithm forms compact and meaningful clusters, effectively grouping data points based on proximity to centroids. Concurrently, players exhibiting distinct attributes are segregated into separate clusters, ensuring a comprehensive categorization (25).

#### 3.1.2 Elbow method and within-sum-of-squares (WSS) metric for player cluster estimation

**Figure 3:**
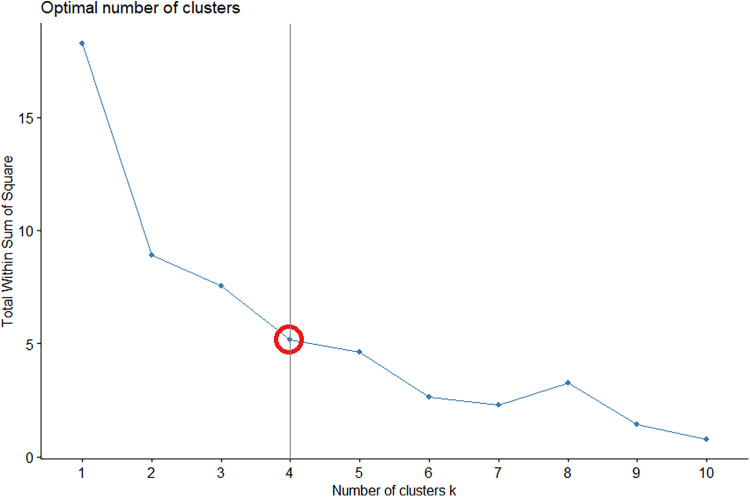
The optimal number of clusters (*k*) was calculated by the elbow method.

**Figure 4:**
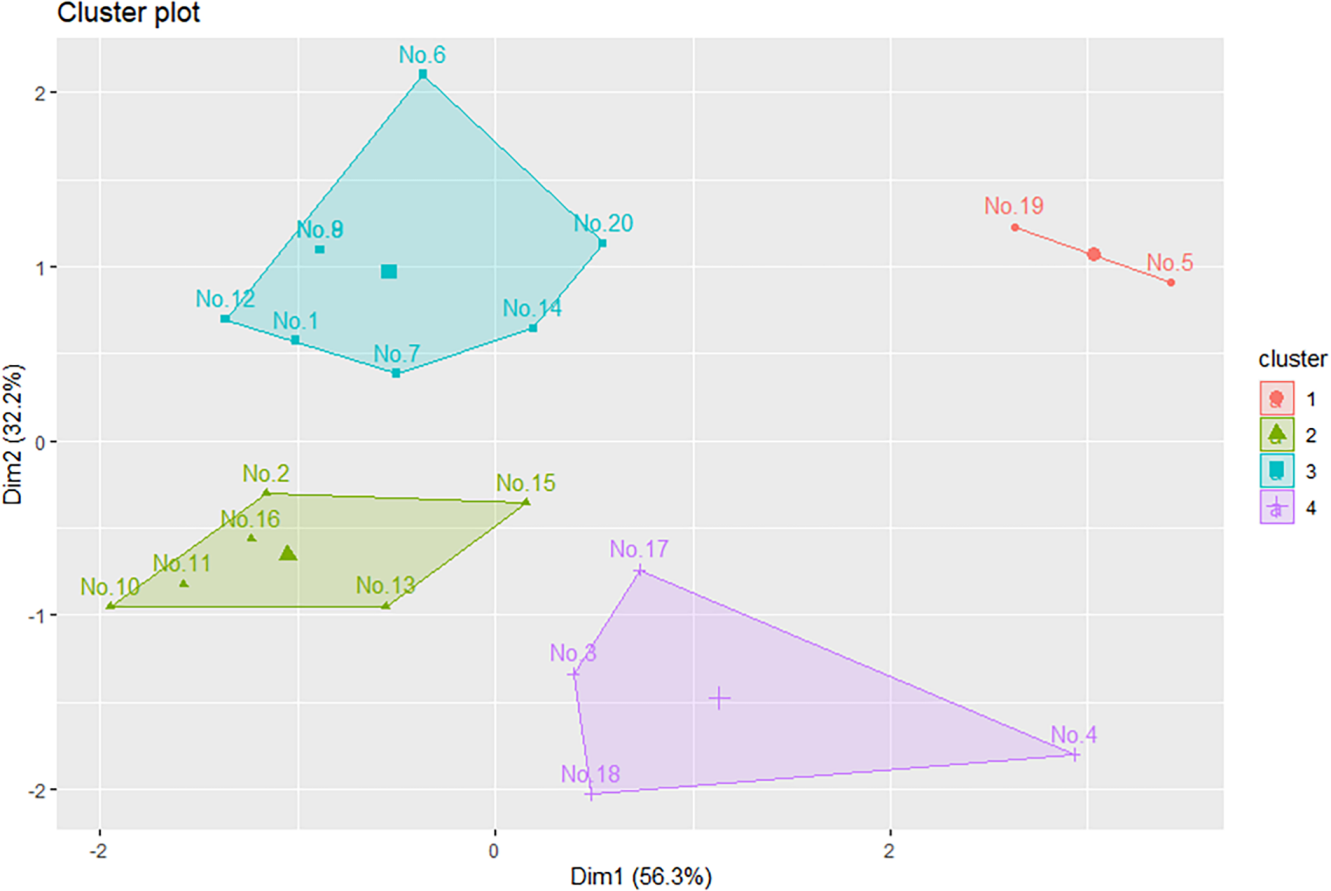
The result of *K*-means cluster analysis.

Prior to conduct the K-means cluster analysis, the elbow method is employed to determine the optimal number of clusters for the dataset (3, 35). The elbow method involves plotting the within-sum-of-squares (WSS) as a measure of dispersion against the number of clusters (*K*). As the value of *K* increases, the WSS index typically decreases, resulting in the characteristic elbow-shaped graph (36). By evaluating the WSS for various values, researchers can identify the point at which additional clusters cease to contribute significantly to the reduction of WSS, thus identifying the optimal number of clusters that yield the most compact and well-defined groupings (36). This approach strengthens the foundation of the K-means clustering analysis and ensures that the resulting clusters are both meaningful and contribute to the robustness of player characterization (37). The elbow method involves tests starting from = 1, 2, 3, …, 10. As shown in Figure 3, the results indicated that the optimal number of clusters for this study was = 4. This conclusion was supported by the observation that at *K* = 4, the performance became stable. Subsequently, the k-means analysis was utilized to separate all players into four clusters, as shown in Figure 4.

## 4 Results

### 4.1 Validity and characterization of player clusters

**Figure 5:**
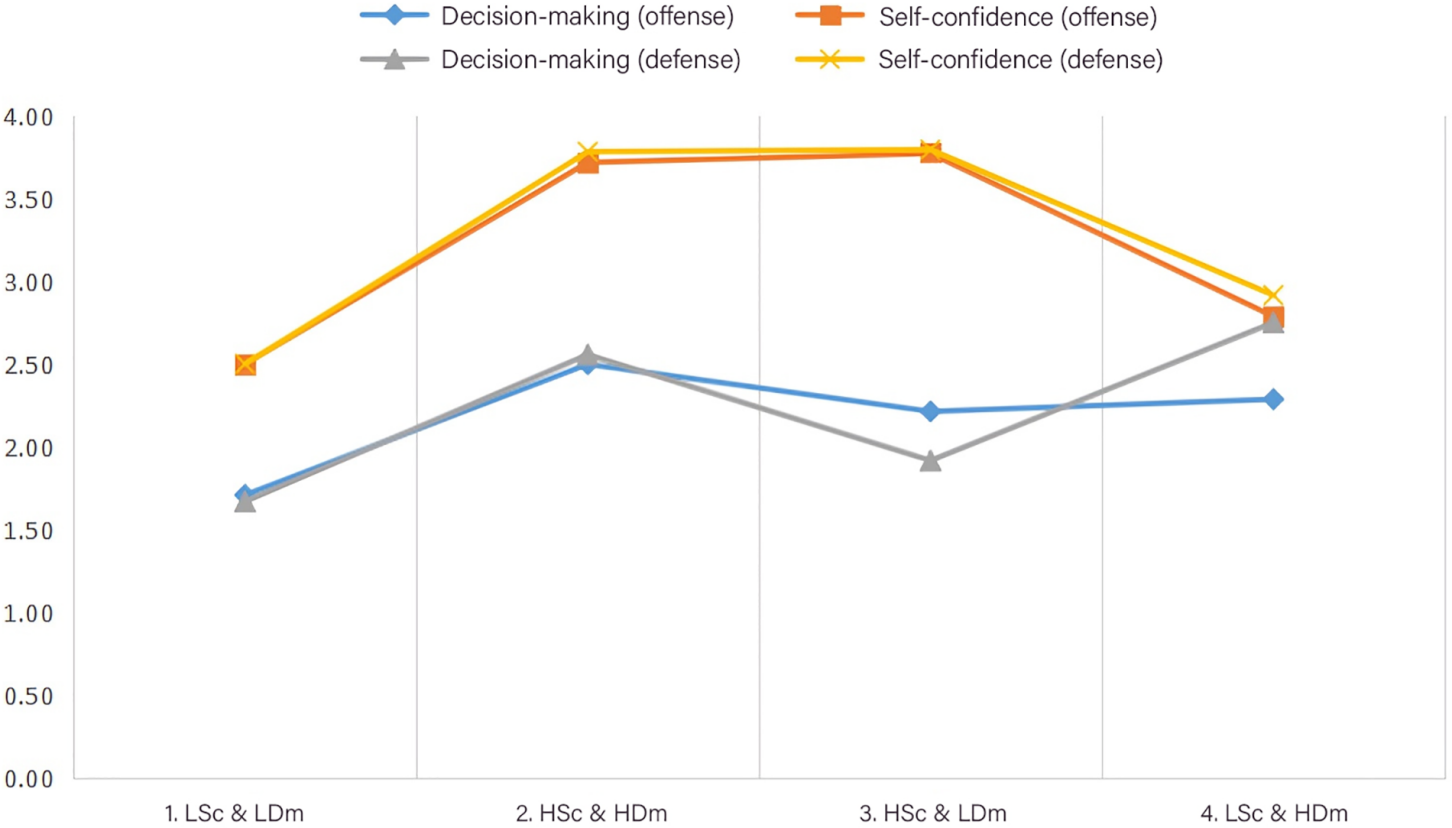
Structure of players segments.

By subjecting the data to ANOVA and *post hoc* Scheffe analysis, statistically significant variations among the four player clusters were presented (*p* < 0.01), as shown in Table 1 and Figure 5. This statistical evidence validated the efficacy of the cluster results, demonstrating that the K-means cluster analysis method used in this study effectively captured and accounted for the distinct characteristics displayed by players within each cluster. In addition, further analyses, including Intraclass Correlation Coefficient (ICC) and regression models, were conducted to explore individual variability and the explanatory power of cluster groupings on key variables. These complementary approaches provided additional validation for the robustness of the clustering method.

**Table 1:** One-way ANOVA results for the four cluster-related factors.

4 clusters (M ± SD)						
	1. LSc & LDm (*n* = 2)	2. HSc & HDm (*n* = 6)	3. HSc & LDm (*n* = 8)	4. LSc & HDm (*n* = 4)	*F*	*p*
Decision-making (offense)	1.71 ± 0.00	**2.50** **±** **0.27**	2.21 ± 0.20	2.29 ± 0.29	5.828	0.007**
Self-confidence (offense)	2.50 ± 0.30	3.71 ± 0.24	**3.77** ± **0.40**	2.79 ± 0.41	12.885	<0.01**
Decision-making (defense)	1.67 ± 0.00	2.56 ± 0.18	1.92 ± 0.15	**2.75** ± **0.32**	26.625	<0.01**
Self-confidence (defense)	2.50 ± 0.24	3.78 ± 0.17	**3.79** ± **0.25**	2.92 ± 0.50	17.453	<0.01**

The highest means are bold. The lowest means are underlined.

* *p* < 0.05 ** *p* < 0.01; M = sample mean; SD = standard deviation.

### 4.2 Labelling of player clusters on self-confidence and decision-making awareness

The cluster characteristics and corresponding labels were summarized based on the results of *K*-means analysis and ANOVA, as shown in Table 2. According to mean (M) and standard deviation (SD) values of 20 players across different four variables, distinct labels were assigned to the four clusters as follows: “LSc & LDm” (Low Self-confidence and Low Decision-making Awareness), “HSc & HDm” (High Self-confidence and High Decision-making Awareness), “HSc & LDm” (High Self-confidence and Low Decision-making Awareness), and “LSc & HDm” (Low Self-confidence and High Decision-making Awareness). These labels effectively characterized the diverse basketball profiles of the players within each cluster, contributing to an understanding of their unique features of decision-making awareness and Self-confidence.

**Table 2:** Cluster labels and characteristics summary.

Clusters	Labels	Characteristics
1	LSc & LDm	The players belonging to the “LSc & LDm” cluster exhibited poor performance across all four variables tested. Notably, their decision-making awareness in both offensive (M ± SD = 1.71 ± 0.00) and defensive (M ± SD = 1.67 ± 0.00) tactics, as well as their Self-confidence in offensive (M ± SD = 2.50 ± 0.30) and defensive (M ± SD = 2.50 ± 0.24) tactics, positioned them at the lowest level among the four clusters. Their performance aligns with that of beginners, displaying a pronounced awareness of their limited competence.
2	HSc & HDm	Players belonging to the “HSc & HDm” cluster demonstrated strong overall performance across all four variables, establishing them as the most proficient group within the team. Notably, their decision-making awareness in both offensive (M ± SD = 2.50 ± 0.27) and defensive (M ± SD = 2.56 ± 0.18) tactics, along with their Self-confidence in offensive (M ± SD = 3.71 ± 0.24) and defensive (M ± SD = 3.78 ± 0.17) tactics, reached high levels among the four clusters. Moreover, their performance remained consistent across different tactics, signifying a stable and high level of basketball ability.
3	HSc & LDm	Players belonging to the “HSc & LDm” cluster displayed a notable discrepancy between their decision-making awareness ability and Self-confidence. Their Self-confidence scores were the highest among the four clusters, indicating a strong belief in their basketball abilities (offense = 3.77 ± 0.40, defense = 3.79 ± 0.25). However, their performance in terms of decision-making awareness fell short (offense = 2.21 ± 0.20, defense = 1.92 ± 0.15), suggesting a lack of proficiency in this aspect. This disparity indicates an overconfident mindset among players of this cluster.
4	LSc & HDm	A mismatch between decision-making awareness and Self-confidence was observed among the players in the “LSc & HDm” cluster. Notably, these players demonstrated commendable performance in decision-making awareness, particularly in the domain of defensive decision-making (M ± SD = 2.75 ± 0.32). However, their Self-confidence scores in offensive (M ± SD = 2.79 ± 0.41) and defensive (M ± SD = 2.92 ± 0.50) were notably low. Encouragement and support are essential for them to enhance their Self-confidence levels.

### 4.3 The discrepancies between and within groups

To further analyze the characteristics of the players in each cluster, *post hoc* Scheffe tests were conducted to perform between-group comparisons, contributing to a more nuanced understanding of their individual profiles and unique features.

As shown in Table 3, the comparison between the LSc&LDm and HSc & HDm revealed the highest level of significance in decision-making awareness for offensive tactics, as evidenced by the *post hoc* Scheffe test (*P* = 0.008; 2 > 1). The results, in conjunction with the ANOVA analysis, highlight those players in the HSc & HDm exhibited the highest level of decision-making awareness in offensive tactics, while those in the LSc&LDm demonstrated the lowest level, with a mean difference of −0.788. However, it is noteworthy that the comparison between the remaining clusters did not exhibit statistical significance, indicating that most players in this team displayed similar decision-making awareness levels in offensive tactics.

**Table 3:** Analysing the differences between the four clusters by decision-making awareness in offensive tactics.

	(I) Player clusters	(J) Player clusters	Mean difference (I-J)	*P*	Scheffe test
Decision-making (offense)	1, LSc & LDm	2, HSc & HDm	−0.788	0.008**	2 > 1
	1, LSc & LDm	3, HSc & LDm	−0.504	0.101	
	1, LSc & LDm	4, LSc & HDm	−0.578	0.082	
	2, HSc & HDm	3, HSc & LDm	0.285	0.212	
	2, HSc & HDm	4, LSc & HDm	0.211	0.598	
	3, HSc & LDm	4, LSc & HDm	−0.074	0.966	

Statistical significance is shown in bold.

* *p* < 0.05 **p < 0.01.

As shown in Table 4, significant differences were observed in the Self-confidence for offensive tactics across most of the cluster comparisons (*p* < 0.01; 2, 3 > 1, 4). Particularly the comparisons between the LSc & LDm and HSc & LDm, and between the HSc & LDm and LSc & HDm, which both displayed the greatest significance (*p* = 0.003). These two comparisons revealed that the Self-confidence scores of the HSc & LDm were the highest among the four clusters, with the comparison between LSc & LDm cluster and HSc & LDm cluster yielding the greatest mean difference of −1.269. In conjunction with the preceding ANOVA analysis, it is evident that both LSc & LDm and LSc & HDm should enhance their confidence levels, which can effectively bolster team morale.

**Table 4:** Analysing the differences between the four clusters by self-confidence in offensive tactics.

	(I) Player clusters	(J) Player clusters	Mean difference (I-J)	*P*	Scheffe test
Self-confidence (offense)	1, LSc & LDm	2, HSc & HDm	−1.215	0.006**	2, 3 > 1; 2, 3 > 4
	1, LSc & LDm	3, HSc & LDm	−1.269	**0**.003**	
	1, LSc & LDm	4, LSc & HDm	−0.288	0.087	
	2, HSc & HDm	3, HSc & LDm	−0.054	0.994	
	2, HSc & HDm	4, LSc & HDm	0.927	**0**.008**	
	3, HSc & LDm	4, LSc & HDm	0.981	**0**.003**	

Statistical significance is shown in bold.

* *p* < 0.05 **p < 0.01.

As shown in Table 5, the decision-making awareness for defensive tactics exhibited significant differences between the four clusters (*P* ≤ 0.001; 2, 4 > 1, 3). Notably, the comparison between the LSc & LDm and the LSc & HDm yielded a mean difference of −1.080, underscoring a substantial variation in decision-making awareness for defensive tactics. However, the significance of the comparisons between the LSc & LDm and the HSc & LDm (*P* = 0.494), as well as between the HSc & HDm and the LSc & HDm (*P* = 0.530), was not as pronounced. As a result, coaches could consider pairing players from these specific clusters during training to potentially increase training efficiency and save training time.

**Table 5:** Analysing the differences between the four clusters by decision-making awareness in defensive tactics.

	(I) Player clusters	(J) Player clusters	Mean difference (I-J)	*P*	Scheffe test
Decision-making (defense)	1, LSc & LDm	2, HSc & HDm	−0.887	0.001**	2, 4 > 1; 2, 4 > 3
	1, LSc & LDm	3, HSc & LDm	−0.248	0.494	
	1, LSc & LDm	4, LSc & HDm	−1.080	**0**.000**	
	2, HSc & HDm	3, HSc & LDm	0.639	**0**.000**	
	2, HSc & HDm	4, LSc & HDm	−0.193	0.530	
	3, HSc & LDm	4, LSc & HDm	−0.833	**0**.000**	

Statistical significance is shown in bold.

* *p* < 0.05 **p < 0.01.

As shown in Table 6, the significant differences were observed in the Self-confidence of defensive tactics, attributed to the lowest levels exhibited by both the “LSc & LDm” and “LSc & HDm” clusters (*p* < 0.01; 2, 3 > 1, 4). The highest significant difference was found in the “LSc & LDm” when compared to both the “HSc & HDm” and “HSc & LDm” (*p* = 0.001). Therefore, a targeted focus on training “LSc & LDm” and “LSc & HDm” is recommended for enhancing Self-confidence in defensive tactics, with particular attention given to the performance of the “LSc & LDm”.

**Table 6:** Analysing the differences between the four clusters by self-confidence in defensive tactics.

	(I) Player clusters	(J) Player clusters	Mean difference (I-J)	*P*	Scheffe test
Self-confidence (defense)	1, LSc & LDm	2, HSc & HDm	−1.280	0.001**	2, 3 > 1; 2, 3 > 4
	1, LSc & LDm	3, HSc & LDm	−1.293	**0**.001**	
	1, LSc & LDm	4, LSc & HDm	−0.415	0.469	
	2, HSc & HDm	3, HSc & LDm	−0.013	1.000	
	2, HSc & HDm	4, LSc & HDm	0.865	**0**.003**	
	3, HSc & LDm	4, LSc & HDm	0.878	**0**.002**	

Statistical significance is shown in bold.

* *p* < 0.05 **p < 0.01.

Based on the above data analysis, within this 20-member basketball team, the performance of decision-making awareness can be ranked as follows, in descending order of performance: 2, HSc & HDm > 4, LSc & HDm > 3, HSc & LDm > 1, LSc & LDm. The “HSc & HDm” exhibited the highest level of performance among the four clusters, while the “LSc & HDm” demonstrated a moderately high level, the “HSc & LDm” showed a lower middle level, and the “LSc & LDm” displayed the lowest level. Furthermore, the performance of Self-confidence can also be ranked as follows, in descending order: 3, HSc & LDm > 2, HSc & HDm > 4, LSc & HDm > 1, LSc & LDm. The “HSc & LDm” demonstrated the highest level of Self-confidence, while the “HSc & HDm” displayed a moderately high level, the “LSc & HDm” exhibited a moderately low level, and the “LSc & LDm” showed the lowest level of Self-confidence. Additionally, within these four clusters, the results also found that the order of clusters based on the number of players, can be ranked as follows, in descending order: HSc & LDm (*n* = 8) > HSc & HDm (*n* = 6) > LSc & HDm (*n* = 4) > LSc & LDm (*n* = 2). The findings of the current study corroborated previous research indicating that in basketball, a prevalent inclination among players is overconfidence (McGraw et al., 2004), which serves as one of the main factors in delineating player characteristics. In conclusion, after comparing the decision-making awareness and Self-confidence of the four clusters in defensive and offensive tactics, the “HSc & LDm” exhibited an overconfident mindset. Conversely, the “HSc & HDm” demonstrated the best performance and maintained a consistent and confident attitude. However, the “LSc & HDm” cluster lacked confidence and needed to be more certain of their abilities. Lastly, the “LSc & LDm” cluster required involvement in a long-term and comprehensive training model.

### 4.4 Consistency and explanatory power of the clusters

To further evaluate the consistency and individual variability of the player clusters, an Intraclass Correlation Coefficient (ICC) analysis was conducted. The ICC results demonstrated a single measure ICC of 0.324 and an average measure ICC of 0.658, indicating moderate to high consistency in player performance across the four variables. These results confirm that the grouping method effectively captures shared characteristics within each cluster.

Additionally, regression analysis was performed to assess the explanatory power of the cluster groupings on key variables. The results indicated that self-confidence in defense had the highest explanatory power (*R*^2^ = 0.608, *p* < 0.001), followed by self-confidence in offense (*R*^2^ = 0.531, *p* < 0.001). Decision-making in offense also showed significant explanatory power (*R*^2^ = 0.378, *p* = 0.004), while decision-making in defense exhibited a weaker association (*R*^2^ = 0.136, *p* = 0.110), as show in Table 7.

**Table 7:** The explanatory power of the cluster groupings on key variables.

	*R* ^2^	*P*
Decision-making (offense)	0.378	0.004**
Decision-making (defense)	0.136	0.110*
Self-confidence (offense)	0.531	0.000***
Self-confidence (defense)	0.608	0.000***

* *p* < 0.5 **p < 0.05 ***p < 0.01.

These analyses offer robust insights into both individual variability and the explanatory significance of the clusters, providing a comprehensive understanding of how psychological factors influence player performance. By combining these complementary statistical methods, the study offers a strong validation of the cluster analysis, further highlighting the importance of tailoring training strategies based on these clusters.

## 5 Discussion

This study analyzed the self-confidence and decision-making awareness of 20 players in both offensive and defensive tactics and used cluster analysis to categorize them into four profiles: (1) LSc & LDm, characterized by lower confidence and limited decision-making ability; (2) HSc & HDm, demonstrating a relatively confident and accurate decision-making process; (3) HSc & LDm, exhibiting overconfidence but relatively weaker decision-making abilities; and (4) LSc & HDm, potentially requiring improved confidence and greater trust in their decision-making abilities.

### 5.1 The self-evaluation matrix

These findings highlight psychological and tactical differences among players on the same team, offering potential insights for basketball training and team management. While the data analysis provides a classification for understanding player profiles, coaches may find it challenging to directly apply these insights to practical management. To bridge this gap, the Self-Evaluation Matrix was developed to help coaches better interpret and utilize these classifications. The matrix is structured around two key dimensions—self-confidence and decision-making—and offers a clear and intuitive framework. The matrix allows coaches to implement more informed management and training decisions, translating complex data into actionable strategies.

**Figure 6:**
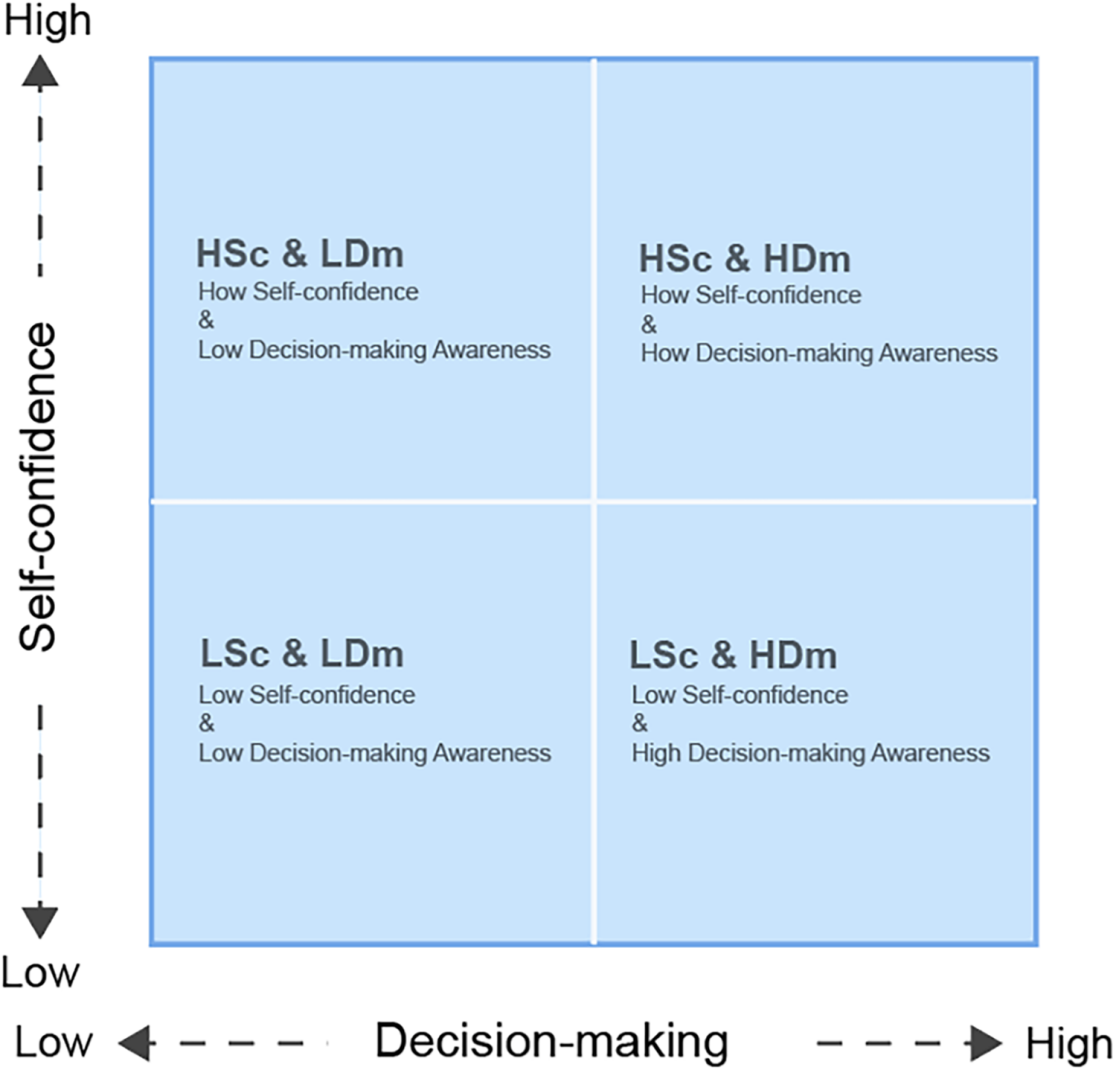
The self-evaluation matrix.

As shown in Figure 6, the Self-Evaluation Matrix categorizes players into four quadrants along two dimensions: self-confidence (vertical axis) and decision-making awareness (horizontal axis). This matrix assists coaches in quickly identifying players’ psychological and tactical characteristics, providing a foundation for customized training strategies tailored to each player's unique profile, thereby enhancing training effectiveness (38). For instance, while players within the four quadrants demonstrate distinct differences, the matrix suggests that those positioned at the corners may exhibit more pronounced traits in self-confidence and decision-making abilities. Further investigation of these extreme characteristics in practice could better inform the design of targeted training interventions for such player profiles.

### 5.2 Practical application of the self-evaluation matrix

#### 5.2.1 The extremes and center of the matrix

**Figure 7:**
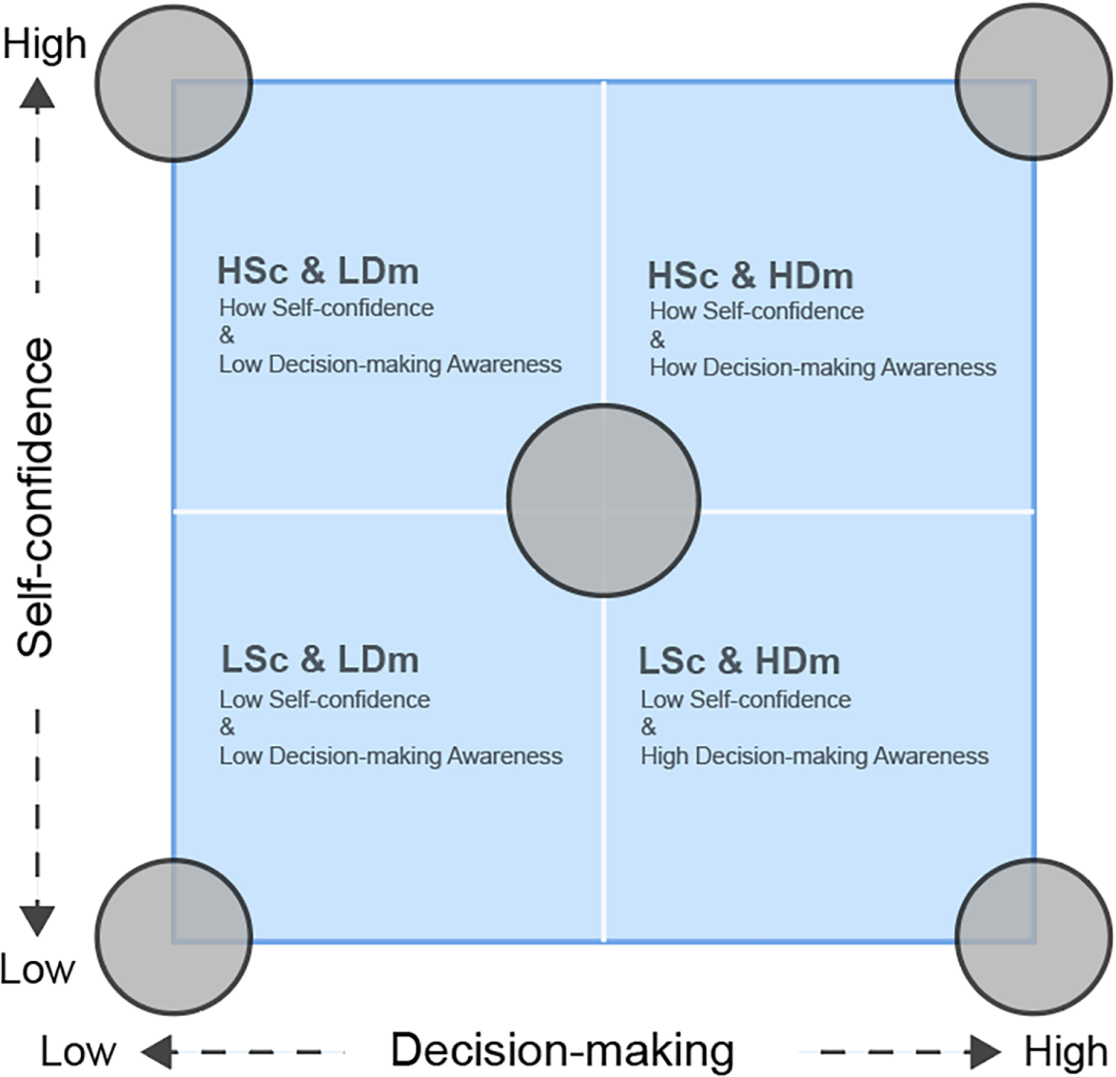
The extremes and center of the matrix.

The model is not only helpful for categorizing players but also enables coaches to design more targeted training strategies based on each player's profile. As shown in Figure 7, players positioned at the extremes of the matrix (in the four corners) exhibit more pronounced characteristics. For example, HSc & LDm players may demonstrate overconfidence, leading to impulsive decisions, particularly in defensive situations. Previous studies suggested that overconfidence can undermine decision-making effectiveness (39). These players could benefit from targeted interventions aimed at improving decision-making under pressure. Conversely, LSc & HDm players exhibit strong tactical awareness but struggle with self-confidence, which may limit their offensive performance. Addressing this lack of confidence through psychological reinforcement strategies may help these players leverage their tactical strengths more effectively in competitive contexts.

This study also uncovered significant discrepancies between the HSc & HDm and LSc & LDm clusters. The two groups of players, LSc & LDm and HSc & HDm, possessed the capacity to accurately evaluate their own performance. For these player groups, the training regimen adhered to conventional patterns for junior and senior players to enhance their performance. Furthermore, these two player groups could be paired to establish a player-support instructional pattern. With HSc & HDm mentoring, LSc & LDm players would have the opportunity to enhance their comprehension of basketball knowledge and cultivate an appropriate mindset. Previous research highlights the benefits of peer teaching in enhancing both decision-making abilities and self-confidence (40).

Following the analysis of the four extreme quadrants, players located near the center of the matrix also should consideration. Players situated in the center generally exhibit a balanced level of self-confidence and decision-making awareness. Although these players tend to perform well across various scenarios, subjecting them to greater challenges—particularly in high-pressure situations—could further enhance their performance. Research indicates that decision-making under pressure can enhance players’ performance in real-game contexts (41).

#### 5.2.2 The overlapping areas of the matrix

**Figure 8:**
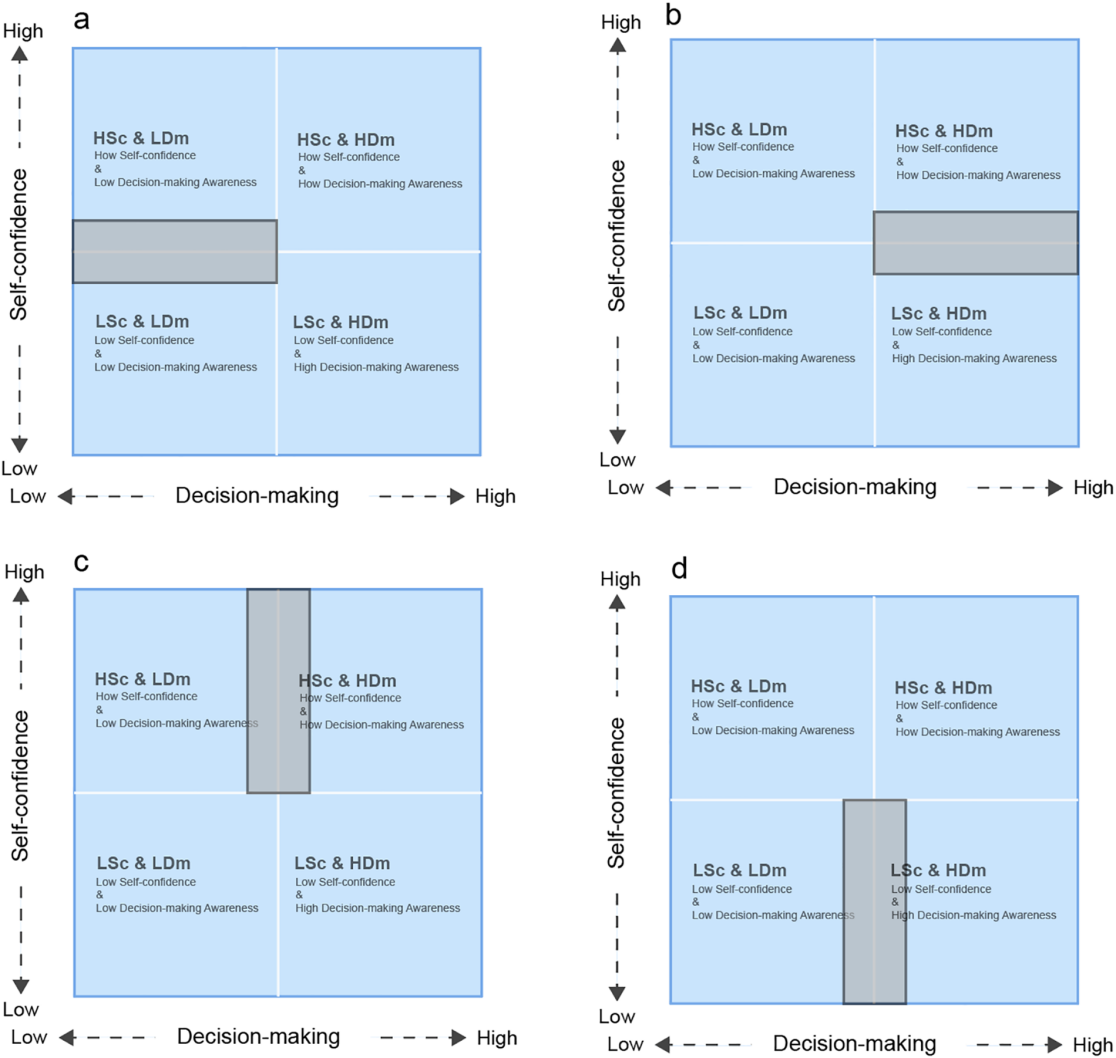
The overlap between HSc & LDm and LSc & LDm **(a)**, the overlap between HSc & HDm and LSc & HDm **(b)**, the overlap between HSc & LDm and HSc & HDm **(c)**, the overlap between LSc & LDm and LSc & HDm **(d)**.

In further analyzing the characteristics of the players, the overlapping areas were considered between different quadrants, which reflect mixed traits in terms of self-confidence and decision-making awareness. For instance, in Figure 8a, the overlap between HSc & LDm and LSc & LDm suggests players with moderate self-confidence but generally weak decision-making abilities. These players may demonstrate fluctuating self-confidence, which does not critically impair their overall performance but can lead to inconsistencies in decision-making under pressure. Coaches should be attentive to these players’ psychological fluctuations, as they may experience challenges in maintaining consistent tactical awareness. In Figure 8b, the overlap between HSc & HDm and LSc & HDm reflects players with similarly moderate self-confidence but more pronounced tactical differences. While some players may display strong decision-making skills, particularly in defense, others lack the confidence needed to capitalize on their abilities. In such cases, psychological reinforcement and exposure to competitive scenarios could help build both confidence and tactical application.

The overlapping regions in Figure 8c between HSc & LDm and HSc & HDm reveal players with high self-confidence but inconsistent decision-making abilities. While their confidence remains stable, their decision-making varies depending on tactical demands. Here, the focus should be on mitigating impulsive decisions while encouraging more tactically sound judgment. Lastly, Figure 8d highlights the overlap between LSc & LDm and LSc & HDm, indicating players with consistently low self-confidence but stronger decision-making capabilities in specific situations, particularly defensive ones. Coaches should prioritize boosting their confidence to ensure they can fully leverage their decision-making strengths.

In these ambiguous areas, coaches must carefully navigate the psychological and tactical complexities to provide tailored interventions. Continuous monitoring of both self-confidence and decision-making fluctuations is essential, as even subtle shifts in psychological readiness can substantially affect performance (42). By refining their understanding of these mixed profiles, coaches can enhance individual and team performance more effectively.

In conclusion, this study contributes to the growing body of research by proposing a conceptual self-evaluation matrix that categorizes players based on self-confidence and decision-making traits. The identification of these four distinct player profiles provides a valuable framework for coaches to better understand the psychological and tactical attributes of their players to enhance performance (43). By leveraging this matrix, coaches can implement more targeted training strategies that address the specific needs of each player profile, particularly those in ambiguous overlapping regions where mixed traits are present. The continuous monitoring of fluctuations in self-confidence and decision-making remains crucial, as even subtle changes can significantly impact performance. Ultimately, the findings underscore the importance of integrating psychological reinforcement, peer-supported teaching patterns, and customized interventions to optimize player performance, fostering both individual growth and enhanced team cohesion.

## 6 Limitations and future research

One limitation of this study was the relatively small sample size, as it was restricted to only one basketball team. Consequently, the data characteristics and findings of the analysis may have been influenced by specific restrictions to that particular team. The generalizability of the methods employed in this study to other sports remains uncertain and requires further investigation. Future research should aim to replicate these findings with larger and more diverse samples to ensure the robustness and applicability of the results.

In addition, while the current study employed researchers to interpret and characterize the player data, it is important to acknowledge that coaches and players may encounter challenges in comprehending the significance of this data in real-time training scenarios. Therefore, the subsequent stage of research should integrate technology and design methods that enables the visualization of player data. By utilizing AI for automated profiling and data visualization, the application of these concepts in the basketball training industry can be simplified and made more accessible for coaches and players on a practical level.

By addressing these limitations and incorporating other research domains, future research in this study has the potential to yield more comprehensive and applicable insights into player performance and decision-making abilities across various sports. These considerations can contribute to the ongoing development of evidence-based strategies and interventions in sports training and performance enhancement.

## 7 Conclusion

This study underscores the significant impact of self-confidence and decision-making awareness on player performance, revealing distinct variations within the same team. Through the development of the self-evaluation matrix, players were classified into four profiles: (1) Low Self-Confidence & Low Decision-Making (LSc & LDm), (2) High Self-Confidence & High Decision-Making (HSc & HDm), (3) High Self-Confidence & Low Decision-Making (HSc & LDm), and (4) Low Self-Confidence & High Decision-Making (LSc & HDm). The matrix not only served to categorize players but also provided an actionable framework for tailoring training strategies based on these profiles. The findings indicated that while LSc & LDm and HSc & HDm players displayed consistent levels of confidence and decision-making abilities, HSc & LDm players tended toward overconfidence, and LSc & HDm players typically underestimated their abilities. The self-evaluation matrix underscores the value of individualized coaching strategies that align with each player's unique profile, optimizing both individual performance and overall team dynamics. Future research should explore the application of this matrix in various competitive environments, assessing the efficacy of targeted training interventions in aligning players’ self-perceptions with their actual abilities. By further refining and implementing the matrix, coaches and practitioners could gain deeper insights into supporting player development and performance optimization in sports settings.

## Data Availability

The original contributions presented in the study are included in the article/Supplementary Material, further inquiries can be directed to the corresponding author.
